# Cardiovascular remodeling during long-term nocturnal home hemodialysis

**DOI:** 10.1007/s10157-014-0992-z

**Published:** 2014-06-04

**Authors:** Tyler Friesen, Davinder S. Jassal, Mike Zhu, Frederick Eng, Claudio Rigatto, Navdeep Tangri, Manish M. Sood, Erin Karlstedt, Sheena Premecz, Paul Komenda

**Affiliations:** 1 Department of Internal Medicine, University of Manitoba, Winnipeg, MB Canada; 2 Section of Nephrology, Department of Internal Medicine, University of Manitoba, Winnipeg, MB Canada; 3 Seven Oaks General Hospital Kidney Health Program, Winnipeg, MB Canada; 4 Institute of Cardiovascular Sciences, St. Boniface General Hospital, University of Manitoba, Winnipeg, MB Canada; 5 Section of Cardiology, Department of Internal Medicine, University of Manitoba, Winnipeg, MB Canada; 6 Department of Radiology, St. Boniface General Hospital, University of Manitoba, Winnipeg, MB Canada; 7 University of Manitoba, Seven Oaks Hospital Renal Program, 2300 Mcphillips Street, 2PD12, Winnipeg, MB R2V 3M3 Canada

**Keywords:** Home hemodialysis, High dose hemodialysis, Left ventricular hypertrophy, Cardiac imaging

## Abstract

**Background:**

Cardiovascular disease is the leading cause of morbidity and mortality in patients with kidney failure. Nocturnal home hemodialysis (NHD) is a form of kidney replacement therapy whereby hemodialysis is performed for at least 6-h overnight, at least 4 days per week. Little is known about the effects of NHD on cardiovascular remodeling as assessed by transthoracic echocardiography (TTE) and cardiac magnetic resonance imaging (CMR).

**Objectives:**

The primary objective of the study was to determine the long-term effects of NHD on cardiovascular remodeling using different imaging modalities over a one-year follow-up.

**Methods and results:**

A total of 11 patients were included in the study (6 males, mean age 48 ± 16 years) between 2009 and 2011 inclusive at a single tertiary care center. All patients underwent TTE and CMR at baseline and after 1 year of NHD. Left ventricular mass index decreased significantly at 1 year by both TTE (152 ± 7–129 ± 8 g/m^2^, *p* < 0.05) and CMR (162 ± 4–124 ± 4 g/m^2^, *p* < 0.05). There was also a significant decrease in both left and right atrial volume as well as in right ventricular mass index over 1 year of follow-up. Diastolic dysfunction, graded from 0 to 4, improved from a baseline grade of 3.4 to 1.2 at 1-year follow-up.

**Conclusions:**

Long-term nocturnal hemodialysis leads to favorable cardiovascular remodeling with a reduction in cavity dimensions, regression of left ventricular hypertrophy, and an improvement in diastolic function, as assessed by both TTE and CMR.

## Background

Cardiovascular disease (CVD) is the most common cause of morbidity and mortality in patients with kidney failure (KF) accounting for nearly half of all deaths [1]. The prevalence of cardiac disease in chronic hemodialysis patients is as high as 80 % [2]. Left ventricular hypertrophy (LVH) is an independent risk factor for cardiac death and is present in greater than 70 % of patients at the initiation of hemodialysis [3]. As such, many outcome studies in hemodialysis patients use LVH as a surrogate marker for cardiovascular events [4–7]. In addition to traditional cardiovascular risk factors including hypertension and diabetes mellitus, patients with chronic kidney disease (CKD) exhibit non-traditional risk factors unique to the uremic environment. These risk factors include elevated pro-inflammatory cytokines, abnormal lipid and bone metabolism, hyperparathyroidism, anemia, volume overload, retention of uremic toxins, and sleep disorders [8–12].

The optimal frequency of hemodialysis has yet to be determined [5]. Most often, patients undergo hemodialysis three times per week for 4 h at a time, although this dialysis dose has rarely been rigorously evaluated in prospective RCT’s. This regimen often results in complications such as large solute and volume shifts causing unstable blood pressures and pulmonary edema. Nocturnal home hemodialysis (NHD) is a form of renal replacement therapy in which hemodialysis is performed in the home for at least 6-h overnight and at least 4 days per week. NHD has not only been shown to cost up to 20 % less than conventional hemodialysis, but it also provides multiple clinical benefits related to blood pressure control and mineral metabolism [13–15].

The cardiovascular effects of NHD, as assessed by transthoracic echocardiography (TTE) and cardiac magnetic resonance (CMR) imaging, have been a subject of recent interest. Chan et al. [6] first reported an improvement in left ventricular mass by TTE in an observational study of 28 patients on NHD over a mean follow-up of 3.4 years. A subsequent randomized controlled trial of 52 patients in Alberta also demonstrated a decrease in LV mass by CMR over a 6-month follow-up [4]. However, a more recent study randomizing 87 patients to conventional hemodialysis vs. NHD did not demonstrate any difference in LV mass as assessed by CMR in NHD patients after 1 year [7]. Little is known, however, about the effects of NHD on both atrial and ventricular remodeling as assessed by TTE and CMR in an incident NHD population…

The primary objective of the study was to determine the effects of NHD on cardiovascular remodeling over a one-year follow-up using both TTE and CMR.

## Methods

### Study population

All patients enrolled in the NHD training program at a single tertiary care center were asked to participate in the study from January 2009 to December 2011 inclusive. For inclusion into the training program, patients were required to be able to perform NHD, have a life expectancy greater than 12 months, and have no reliable expectation of receiving a kidney transplant within 12 months. The study protocol was approved by the University of Manitoba research ethics board (REB protocol number H2008:279).

### Study protocol

Upon enrollment into the NHD training program, patients underwent 6–10 weeks of one-on-one training with a nurse. The patients went on to perform daytime home hemodialysis for 1–4 weeks, followed by overnight extended hours hemodialysis. All patients had TTE and CMR studies performed at baseline and after 1 year of NHD. All cardiac imaging parameters were performed the day following an overnight hemodialysis run when patients are closest to their prescribed dry weight. Demographic, clinical, and laboratory data were collected at baseline. Hematology and chemistry laboratory values were obtained monthly both pre- and post-dialysis. Parathyroid hormone and lipid profiles were measured every 3 months.

### Echocardiography

Transthoracic echocardiography was performed using a standard echocardiography machine (GE Vivid 7, Milwaukee, WI, USA) at baseline and 12-month follow-up. Cardiac chamber dimensions and function were determined according to the American Society of Echocardiography guidelines [16]. Transmitral left ventricular (LV) filling velocities were measured at the tips of the mitral valve leaflets using the apical four-chamber view and pulsed-wave Doppler. Manual tracing of the transmitral LV filling signal was performed to obtain peak early (E) and late (A) transmitral velocities, E/A ratio, and E wave deceleration time. Tissue Doppler-derived indices at the lateral mitral annulus included systolic velocities (S’), early diastolic velocities (E’), and late diastolic velocities (A’). Finally the E/E’ index was determined. Echocardiographic analysis was performed by two independent reviewers, blinded to the clinical data, using dedicated computer software (EchoPAC, version 110.0.0, GE Medical, Milwaukee, WI, USA).

### Cardiac magnetic resonance imaging

All patients underwent a CMR study at baseline and at 12 months following initiation of NHD. All CMR studies were performed using a 1.5-T Siemens Scanner (Magnetom Sonata, Siemens Medical Systems, Erlangen, Germany). Cardiac parameters of interest included chamber dimensions, volumes, and systolic function which were analyzed in accordance with guidelines of the Society for Cardiovascular Magnetic Resonance [17]. End-systolic and end-diastolic volumes of the left and right ventricle were obtained using manual tracing of ventricular walls in multiple short axis slices. End diastole was defined as the slice in which the ventricle was at its largest volume, while end systole was defined as the slice with the smallest volume. Stroke volume (SV) was calculated as the difference between the end-diastolic volume (EDV) and end-systolic volume (ESV). Left and right ventricular mass were determined using the summation of slices method [18]. Endocardial and epicardial borders of the left and right ventricle, excluding papillary muscles, were manually traced in each image slice used to calculate EDV and ESV. Myocardial volume was calculated by multiplying these values by slice thickness. Myocardial mass was then determined by multiplying each volume by 1.05 g/cm^3^. Analysis of CMRs was conducted by two independent reviewers, blinded to the clinical data, using dedicated computer software (CMR42, version 1.0.0, Circle Cardiovascular Imaging, Calgary, AB, Canada).

### Statistical analysis

All parametric data were reported as mean ± standard deviation (SD). Categorical data were reported as “n” (percentage). The Mann–Whitney *U* test was used to measure the intra- and inter-observer variability for LV end-diastolic volume and LV mass for both imaging modalities. Statistical significance was defined as *p* < 0.05. SAS version 8.01 (SAS Institute Inc., Cary, North Carolina) was used to perform the analysis.

## Results

### Study population

A total of 11 patients (mean age 48 ± 16 years) were enrolled in the study, of which 6 were male (Table 1). Ten patients underwent conventional, thrice-weekly facility-based hemodialysis at baseline (prior to enrollment), while one patient performed home peritoneal dialysis. The most frequent etiology of kidney failure was glomerulonephritis (55 %), followed by diabetic nephropathy (18 %) and polycystic kidney disease (18 %). Cardiac comorbidities included hypertension (63 %), ischemic heart disease (27 %), diabetes mellitus (36 %), and valvular heart disease (9 %).

**Table 1:** Baseline characteristics of the NHD patient population

Characteristic	Patient population (*n* = 11)
Age (years), mean ± SD	48 ± 16
Male	6
Female	5
Ethnicity	
Caucasian	7 (64 %)
First Nations	3 (27 %)
Asian	1 (9 %)
BMI (kg/m^2^), mean ± SD	23 ± 4
Prior renal transplant	4 (36 %)
Baseline dialysis modality	
Hemodialysis	10 (91 %)
Peritoneal dialysis	1 (9 %)
Vascular access	
AV fistula	10 (91 %)
Tunneled catheter	1 (9 %)
Cause of ESRD	
Diabetic nephropathy	2 (18 %)
Glomerulonephritis	6 (55 %)
Polycystic kidney disease	2 (18 %)
Unknown	1 (9 %)
Comorbidities	
Hypertension	7 (64 %)
Ischemic heart disease	3 (27 %)
Diabetes mellitus	4 (36 %)
Valvular heart disease	1 (9 %)
Smoker	1 (9 %)
Weight	
Dry weight (kg) 0 months, mean ± SD	65.62 ± 14.02
Dry weight (kg) 12 months, mean ± SD	66.23 ± 14.50
Interdialytic weight gain (kg) 0 months, mean ± SD	1.74 ± 1.18
Interdialytic weight gain (kg) 12 months, mean ± SD	1.54 ± 0.77

### Echocardiography

**Fig. 1:**
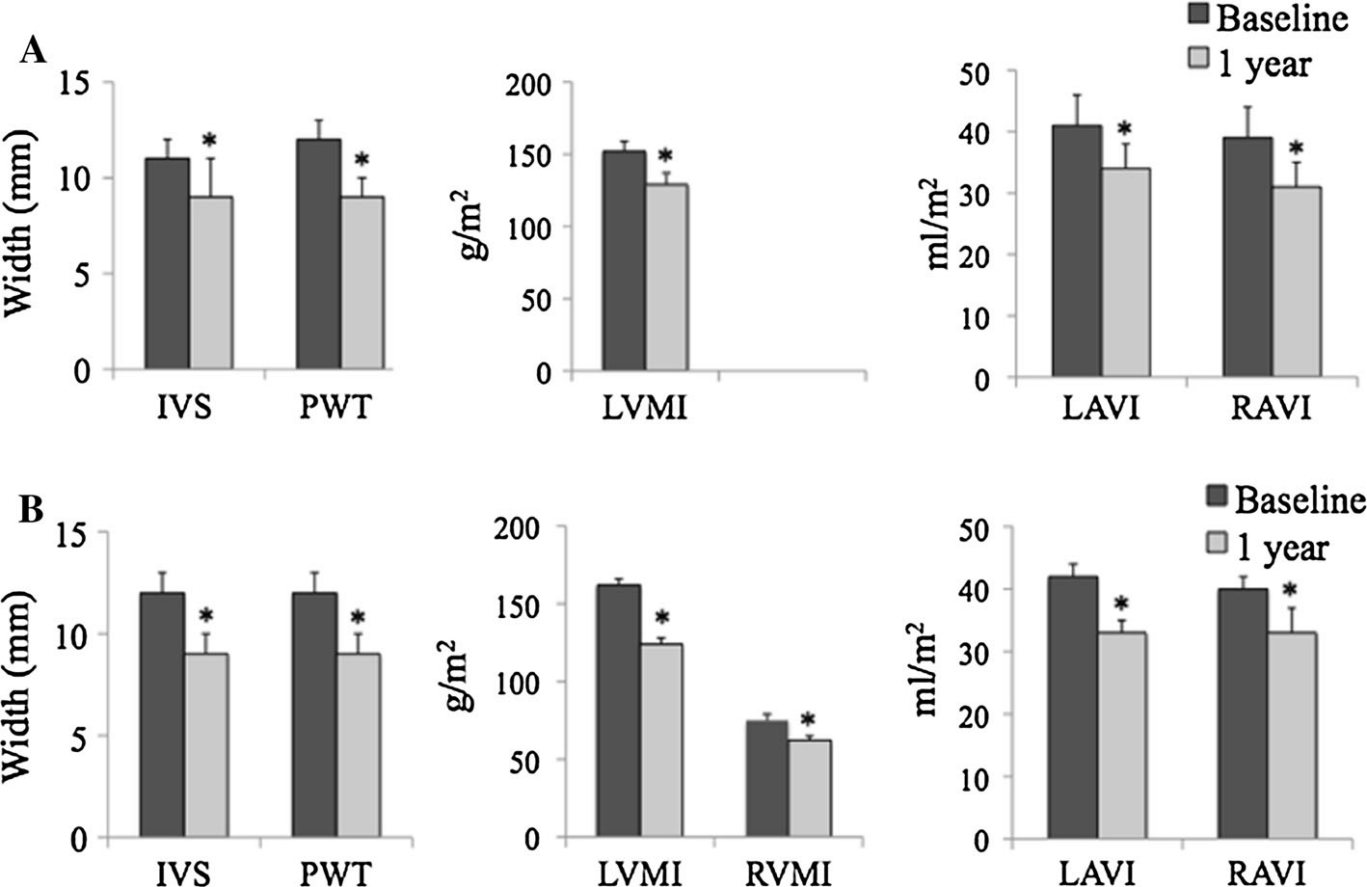
Cardiac dimensions by transthoracic echocardiography (TTE, A) and cardiac magnetic resonance imaging (CMR, B) at baseline and after 1 year of nocturnal home hemodialysis (NHD). *IVS* interventricular septum, *PWT* posterior wall thickness, *LVMI* left ventricular mass index, *RVMI* right ventricular mass index, *LAVI* left atrial volume index, *RAVI* right atrial volume index. * *p* < 0.05

The echocardiographic measurements for the study population are listed in Table 2. There was a significant reduction in interventricular septal (IVS) thickness (11 ± 1 to 9 ± 2 mm, *p* < 0.05) as well as in posterior wall thickness (PWT), (from 12 ± 1 to 9 ± 1 mm, *p* < 0.05) by TTE over the one-year follow-up. In addition, there was a 15 % reduction in left ventricular mass index (LVMI, 152 ± 7 to 129 ± 8 g/m^2^, *p* < 0.05; Fig. 1) on long-term NHD. There were significant reductions in both left atrial volume index (LAVI, 41 ± 5 to 34 ± 4 ml/m^2^, *p* < 0.05) and right atrial volume index (RAVI, 39 ± 5 to 31 ± 4 ml/m^2^, *p* < 0.05). Finally, diastolic dysfunction improved from a baseline grade of 3.4 to 1.2 after one-year follow-up (*p* < 0.05) as shown in Table 3. There was a decrease in the E wave velocity with no change in the A wave velocity over time, resulting in a decrease in the E/A ratio over 1-year follow-up. The LV filling pressures, as reflected by the E/E’, also improved over time. There were no significant changes in left ventricular end-systolic and end-diastolic dimensions, nor any change in left ventricular ejection fraction (LVEF) or cardiac output (CO) at one-year follow-up. There was good intra-observer and inter-observer variability for the measurement of LVMI (Table 4).

**Table 2:** Cardiac chamber parameters by TTE and CMR at baseline and 1-year follow-up in total population *(n* = 11*)*

	TTE			CMR		
	Baseline	1 year follow-up	*p*	Baseline	1 year follow-up	*p*
LV parameters						
LVEDD (mm)	45 ± 4	46 ± 4	0.86	46 ± 1	47 ± 2	0.82
LVESD (mm)	31 ± 2	32 ± 3	0.83	31 ± 3	32 ± 3	0.71
LVEDV (mL)	96 ± 9	98 ± 10	0.85	99 ± 6	100 ± 7	0.82
LVESV (mL)	29 ± 7	30 ± 6	0.77	30 ± 5	32 ± 5	0.81
IVS (mm)	**11** **±** **1**	**9** **±** **2**	**<0.05**	**12** **±** **1**	**9** **±** **1**	**<0.05**
PWT (mm)	**12** **±** **1**	**9** **±** **1**	**<0.05**	**12** **±** **1**	**9** **±** **1**	**<0.05**
SV (mL)	63 ± 11	65 ± 7	0.68	64 ± 6	66 ± 8	0.76
HR (bpm)	70 ± 7	74 ± 9	0.62	73 ± 8	75 ± 6	0.82
CO (L/min)	4.2 ± 0.9	4.6 ± 0.7	0.54	4.4 ± 0.2	4.5 ± 0.4	0.81
LVEF (%)	69 ± 8	70 ± 5	0.76	64 ± 3	65 ± 4	0.75
LV mass index (g/m^2^)	**152** **±** **7**	**129** **±** **8**	**<0.05**	**162** **±** **4**	**124** **±** **4**	**<0.05**
RV parameters						
RVEDD (mm)	33 ± 5	34 ± 4	0.82	34 ± 5	35 ± 3	0.76
RVEF (%)	–	–	–	63 ± 3	64 ± 3	0.80
RV mass index (g/m^2^)	**–**	**–**	**–**	**75** **±** **4**	**62** **±** **3**	**<0.05**
RV FAC (%)	45 ± 4	46 ± 5	0.76	–	–	–
TAPSE (mm)	3.2 ± 0.3	3.2 ± 0.4	0.91	–	–	–
PASP (mmHg)	32 ± 3	33 ± 4	0.72	–	–	–
Atrial parameters						
LA diameter (mm)	32 ± 3	33 ± 4	0.72	32 ± 2	33 ± 3	0.81
LA volume index (mL/m^2^)	**41** **±** **5**	**34** **±** **4**	**<0.05**	**42** **±** **2**	**33** **±** **2**	**<0.05**
RA volume index (mL/m^2^)	**39** **±** **5**	**31** **±** **4**	**<0.05**	**40** **±** **2**	**33** **±** **4**	**<0.05**

Bold values indicate that *p* < 0.05 are significant compared to baseline

**Table 3:** Diastolic parameters by TTE at baseline and 1-year follow-up in total population (*n* = 11)

	Baseline	1 year follow-up	*p*
Diastolic grade			
E wave velocity (m/s)	1.4 ± 0.3	0.7 ± 0.3	<0.05
A wave velocity (m/s)	0.4 ± 0.3	0.5 ± 0.3	<0.05
E/A ratio	3.5 ± 0.2	1.4 ± 0.2	<0.05
Deceleration time (m s)	195 ± 40	208 ± 25	<0.05
Diastolic grade	3.4	1.2	<0.05
TDI parameters (LV)			
Lateral S’ (cm/s)	9.8 ± 0.3	10.2 ± 0.4	0.77
Lateral E’ (cm/s)	8.2 ± 0.5	8.2 ± 0.4	0.91
Lateral A’ (cm/s)	7.9 ± 0.6	8.0 ± 0.3	0.82
Medial S’ (cm/s)	9.6 ± 0.7	9.4 ± 0.5	0.81
Medial E’ (cm/s)	8.0 ± 0.5	8.3 ± 0.6	0.83
Medial A’ (cm/s)	8.5 ± 0.4	8.1 ± 0.3	0.76
E/E’	17 ± 1	8 ± 1	<0.05
TDI parameters (RV)			
Lateral S’	9.3 ± 0.4	9.1 ± 0.3	0.80
Lateral E’	8.1 ± 0.3	8.0 ± 0.2	0.77
Lateral A’	7.9 ± 0.3	7.7 ± 0.4	0.82

Data are expressed as mean ±SD

*E wave* early diastolic filling, *A wave* late diastolic filling, *TDI* tissue Doppler imaging, *S’* systolic myocardial velocity, *E’* early diastolic myocardial velocity, *A’* late diastolic myocardial velocity

* *P* < 0.05, 1-year follow-up vs. baseline

**Table 4:** Intra-observer and inter-observer variability for LV mass index (*n* = 11)

	Intra-observer		Inter-observer	
	Absolute	%	Absolute	%
LV mass index (g/m^2^)				
TTE	12.2 ± 3.4	10.3 ± 4.2	11.1 ± 3.3	9.5 ± 3.9
CMR	7.6 ± 3.1	5.7 ± 1.8	8.4 ± 2.2	5.5 ± 1.4

### Cardiac magnetic resonance imaging

As compared to TTE, there were similar reductions in IVS thickness (12 ± 1–9 ± 1 mm, *p* < 0.05) and PWT (12 ± 1–9 ± 1 mm, *p* < 0.05) by CMR (Table 2). There was a significant reduction in LVMI by 23 % by CMR (162 ± 4–124 ± 4 g/m^2^, *p* < 0.05). In addition, there were significant decreases in LAVI (42 ± 2–33 ± 2 ml/m^2^, *p* < 0.05) and RAVI (40 ± 2–33 ± 4 ml/m^2^, *p* < 0.05) with narrower confidence intervals using CMR as compared to TTE (Table 2; Fig. 1). Moreover, right ventricular mass index (RVMI) showed significant regression after one-year follow-up (75 ± 4–62 ± 3 g/m^2^, *p* < 0.05). There were no significant changes in left ventricular end-systolic and end-diastolic dimensions, LVEF, nor CO at one-year follow-up using CMR. There was good intra-observer and inter-observer variability for the measurement of LVMI (Table 4).

### Secondary endpoints

Data regarding blood pressure, mineral metabolism, anemia and albumin levels are summarized in Table 5. Overall, there were no significant differences in any of these parameters after 1 year of NHD.

**Table 5:** Secondary endpoints at baseline and after 1 year of NHD (*n* = 11)

Parameter	Baseline (mean ± SD)	One-year follow-up (mean ± SD)	*p*
Pre-dialysis SBP (mmHg)	126.5 ± 19.6	122.3 ± 18.6	0.66
Pre-dialysis DBP (mmHg)	74.9 ± 11.9	68.6 ± 7.3	0.23
Pre-dialysis serum calcium (mmol/L)	2.39 ± 0.22	2.42 ± 0.15	0.74
Pre-dialysis serum phosphate (mmol/L)	1.48 ± 0.29	1.46 ± 0.38	0.87
Hemoglobin (g/L)	112 ± 11.5	113.5 ± 11.1	0.76
Albumin (g/L)	38.9 ± 1.8	38.2 ± 3.0	0.51
Parathyroid hormone	379 ± 232	249 ± 169	0.18

## Discussion

Cardiovascular disease is the leading cause of death in patients with kidney failure on dialysis. Although NHD is associated with significant clinical benefits in this patient population, its effects on cardiovascular remodeling remain unclear. While previous studies have investigated the effect of NHD on left ventricular mass alone by either TTE or CMR, the results have been conflicting. This is the first study to comprehensively evaluate cardiac remodeling using both TTE and CMR in an incident cohort of patients who have converted from conventional thrice-weekly hemodialysis to NHD. Following one year of compliant use of NHD, there was an improvement in biventricular mass index, biatrial volume index, and the degree of diastolic dysfunction in our ESRD population. Left ventricular hypertrophy is very common in kidney failure, affecting more than 70 % of patients at initiation of hemodialysis [3]. In addition to traditional risk factors for the development of LVH including hypertension, age, and valvular heart disease, there are a number of risk factors unique to patients with chronic kidney disease (CKD). Hemodynamic abnormalities due to volume overload, anemia, vascular calcification, and the presence of an arterio-venous fistula are important determinants of LV mass [19]. Additional contributing factors include hyperphosphatemia, hyperparathyroidism, and hypovitaminosis D [19].

In the current study, we demonstrated significant regression of LVH after 1 year of NHD, by both TTE and CMR. Two previous randomized studies of NHD using CMR alone have shown conflicting results with respect to regression of LVH [4, 7]. While Culleton et al. [4] demonstrated an 8 % reduction in LVMI by CMR after 6 months of NHD, a more recent study by Rocco et al. [7]. did not find any difference in LVMI by CMR in a larger cohort of patients after 1 year of NHD. Our study population was slightly younger, with a lower prevalence of hypertension compared to these two trials. A unique finding of our study was that the regression of LVH was not associated with any improvement in blood pressure control. This could be due to the small sample size or the low prevalence of hypertension in our study population. It is plausible that factors other than blood pressure play an important role in LV remodeling in the ESRD population on NHD.

Regression of LVH has been shown to improve systolic function, and reduce the risk of ventricular arrhythmias and atrial fibrillation [20–22]. Moreover, in patients with and without kidney failure, regression of LVH is associated with decreased all-cause mortality, rendering this a valid surrogate health outcome in this population [23, 24].

Left atrial enlargement is a common echocardiographic finding in patients with ESRD, affecting greater than 40 % of asymptomatic patients with stage 3 to 5 CKD [25]. Multiple factors may lead to LA enlargement including extracellular volume overload, LV dysfunction, LVH and valvular heart disease, all of which are common in ESRD patients [26]. Observational studies in dialysis patients have shown that LA enlargement is significantly correlated with mortality risk, independent of LVMI and LV ejection fraction [26, 27]. Right atrial enlargement has also been shown to be an independent risk factor for the development of atrial fibrillation [28]. To our knowledge, this is the first TTE and CMR study to report the effect of NHD on atrial size. In our study, there was a significant decrease in RAVI and LAVI by TTE and CMR after 1 year of NHD. These results suggest that atrial remodeling may be reversed with NHD, thus potentially lowering the risk of future cardiovascular complications, including atrial rhythm disturbances in the CKD population.

Diastolic dysfunction is an independent predictor of mortality and is the most common echocardiographic finding in asymptomatic dialysis patients [19, 29]. Diastolic dysfunction is strongly associated with hypertension, LVH, coronary artery disease, and diabetes mellitus, all of which are common in patients with ESRD [19]. The increase in left ventricular stiffness causes a shift of the pressure–volume curve to the left, leading to an increased sensitivity to changes in LV volume. Small increases in LV volume can lead to pulmonary congestion while small decreases in LV volume can lead to hypotension [19]. While previous studies have shown regression of LVH in ESRD patients who convert to NHD [4, 6], no study has reported the effect of NHD on diastolic function. This study is the first to show a significant improvement in diastolic dysfunction from a grade of 3.4 to 1.2 after 1 year of NHD with an improvement in overall LV filling pressures. While regression of diastolic dysfunction has been associated with LVH regression in prior studies, it is not known whether this leads to improved survival or a reduction in cardiovascular events [20, 30].

There are several important limitations of our study. First of all, due to the limited sample size, our study may have been underpowered to detect differences in our secondary endpoints. Secondly, this was an observational cohort study. While randomized controlled study design is considered the gold standard, it may be difficult to ethically justify randomizing patients to a modality of renal replacement therapy considered by many to be inferior either in terms of clinical parameters, costs or most likely patient preference. Difficulty in randomizing patients to receive home nocturnal hemodialysis versus conventional facility-based hemodialysis in the contemporary era of increased availability for home hemodialysis has been reported [7]. Finally, our study reported surrogate outcomes for cardiovascular endpoints such as morbidity and mortality. To date, no studies have reported improvement in cardiovascular outcomes with NHD; however, the one study that reported cardiovascular outcomes was likely underpowered to detect a difference [7]. An adequate study of the effect of NHD on cardiovascular outcomes would need to include a large number of patients over a long follow-up period, which is logistically challenging.

## Conclusions

Long-term nocturnal hemodialysis leads to favorable cardiovascular remodeling as measured by a number of parameters and two imaging modalities; TTE and CMR. After 1 year of NHD, patients experience a regression of LVH as well as an improvement in diastolic dysfunction, atrial enlargement, and right ventricular mass index.
